# Yellow fever outbreak in Brazil: the puzzle of rapid viral spread and challenges for immunisation

**DOI:** 10.1590/0074-02760180278

**Published:** 2018-09-03

**Authors:** Cristina Possas, Ricardo Lourenço-de-Oliveira, Pedro Luiz Tauil, Francisco de Paula Pinheiro, Alcides Pissinatti, Rivaldo Venâncio da Cunha, Marcos Freire, Reinaldo Menezes Martins, Akira Homma

**Affiliations:** 1 Fundação Oswaldo Cruz-Fiocruz, Bio-Manguinhos, Rio de Janeiro, RJ, Brasil; 2 Fundação Oswaldo Cruz-Fiocruz, Instituto Oswaldo Cruz, Rio de Janeiro, RJ, Brasil; 3 Universidade de Brasília, Faculdade de Medicina, Brasília, DF, Brasil; 4 Centro de Primatologia do Rio de Janeiro, Instituto Estadual do Ambiente, Guapimirim, RJ, Brasil; 5 Fundação Oswaldo Cruz-Fiocruz, Campo Grande, MS, Brasil

**Keywords:** yellow fever, vaccine, eco-social factors, human immunization, monkey immunization, vaccine production, Aedes aegypti

## Abstract

We discuss the complex eco-social factors involved in the puzzle of the unexpected rapid viral spread in the ongoing Brazilian yellow fever (YF) outbreak, which has increased the reurbanisation risk of a disease without urban cases in Brazil since 1942. Indeed, this rapid spatial viral dissemination to the Southeast and South regions, now circulating in the Atlantic Forest fragments close to peri-urban areas of the main Brazilian megalopolises (São Paulo and Rio de Janeiro) has led to an exponential increase in the number of yellow fever cases. In less than 18 months, 1,833 confirmed cases and 578 deaths were recorded most of them reported in the Southeast region (99,9%). Large epizooties in monkeys and other non-human primates (NHPs) were communicated in the country with 732 YF virus (YFV) laboratory confirmed events only in the 2017/2018 monitoring period. We also discuss the peculiarities and similarities of the current outbreak when compared with previous great epidemics, examining several hypotheses to explain the recent unexpected acceleration of epizootic waves in the sylvatic cycle of the YFV together with the role of human, NHPs and mosquito mobility with respect to viral spread. We conclude that the most feasible hypothesis to explain this rapidity would be related to human behavior combined with ecological changes that promoted a significant increase in mosquito and NHP densities and their contacts with humans. We emphasize the urgent need for an adequate response to this outbreak such as extending immunisation coverage to the whole Brazilian population and developing novel strategies for immunisation of NHPs confined in selected reserve areas and zoos. Finally, we stress the urgent need to improve the quality of response in order to prevent future outbreaks and a catastrophic reurbanisation of the disease in Brazil and other South American countries. Continuous monitoring of YFV receptivity and vulnerability conditions with effective control of the urban vector *Aedes aegypti* and significant investments in YF vaccine production capacity and research and development for reduction of adverse effects are of the highest priority.

Brazilian authorities and scientists have reported with perplexity the worst outbreak of sylvatic yellow fever (YF) in the last 80 years in the country with an exponential increase in the number of confirmed cases and deaths in humans and epizootics in non-human primates (NHPs). The ongoing outbreak spread from November 2016 into forest areas contiguous to the country’s largest megalopolises in the Southeast region, such as São Paulo and Rio de Janeiro, significantly increasing the risk of reurbanisation of the disease. There are complex eco-social determinants of this rapid YF viral spread, but also policy constraints and erroneous strategies certainly have contributed to accelerate and aggravate this epidemiological scenario. The goal of minimising the risk of resurgence of urban YF in Brazil, a disease eliminated in 1942, is theoretically achievable and would require simply immunisation of all the Brazilian population with the highly effective YF vaccine preferably combined with reduction of *Aedes aegypti* infestation indexes. Nevertheless, from a practical perspective, the pathway to achieve this goal has been permeated by erroneous policies and strategies: (1) negligence in early detection and reporting of epizootics in YF virus (YFV) emergence as well as surveillance in non-endemic areas in 2016; (2) delayed immunisation response from the Brazilian authorities with very low vaccine coverage which has resulted in an extremely high incidence of the disease in rural and peri-urban areas, thus leading to an exponential increase in the demand for vaccines; (3) concentration of YF immunisation on children, leaving uncovered the adult population which is generally the most affected by the sylvatic YF outbreaks; (4) delay in reaching and providing YF immunisation for rural workers living close to forest areas and eco-tourists, more exposed to the YFV; (5) failure in social communication on the effectiveness of the YF vaccine and dealing with vaccination refusal consequent to an exacerbated perception of its adverse effects; and finally (6) very low sanitation coverage (< 50% of the Brazilian population) together with failure in *Ae*. *aegypti* and *Ae. albopictus* control, enhancing the risk of YFV re-urbanisation.

We provide epidemiological information and scientific evidence on the complexity of this scenario and emphasize the urgent need to intensify human immunisation coverage by increasing vaccine availability and implementing campaigns with solid communication interventions to preclude stagnation of coverage levels. We also emphasize the urgency to develop innovative strategies, as vaccinating NHPs, especially endemic and endangered species confined in preservation areas, avoiding their extinction and reducing their potential role as local YFV amplifiers. Although controversies among primatologists on the feasibility of NHP immunisation have emerged due to potential difficulties in capturing and/or injecting vaccine in free-living animals in the open field owing to their high mobility, novel technological strategies for monkey immunisation, preserving biodiversity in Brazil, should be conceived and evaluated. Some of these novel strategies are suggested herein.

YF epidemiology: rapid viral spread and emergency

There has historically been a consensus among scientists and policy makers in Brazil that YFV transmission would probably remain confined to sylvatic cycles in areas with low population densities and with a low number of annual cases as in the past since the last urban case in 1942. Nevertheless, environmental, eco-social and human behavioral changes in the last decades have created conditions for an exponential increase in YF cases and case fatalities in the country during the ongoing outbreak (Fig. 1), in which the risk for reurbanisation of the disease has significantly amplified. Indeed, since November 2016, after decades of silence, the YFV has spread into the coastal Atlantic Forest zones and rapidly moved into the Southeast and South of the country, reaching in less than one year four of the most populous Brazilian states (Minas Gerais, São Paulo, Rio de Janeiro and Espírito Santo) whose residents had not been included in the YFV vaccination program. The low vaccination coverage of potential risk areas in these highly populated states has resulted in a sharp increase in the number of YF cases in the country: 1,833 confirmed cases and 578 deaths were recorded in less than 18 months. A total of 1257 confirmed cases and 394 deaths were reported up to EW17 (April 28 2018) in the monitoring period 2017/2018 (July 1 to June 30).^1^ This number is much higher than that reported for the same period of 2016/2017 with 576 confirmed cases including 184 deaths and contrasts with the very low number of human cases and epizootics in the respective monitoring period in 2015 when three YF human cases were confirmed in the country with two deaths. Finally, it should be noted that early reports of YF cases in the 1930-1950s in Brazil were concentrated on case fatalities, so examining the evolution of lethality rate up to 2017 would be misleading.

Moreover, it should be emphasised that evidences of the emergence of the current YFV transmission wave were recorded as early as in July 2014, with confirmed epizooties in the transition area between the Amazon and *Cerrado*, a savanna-like biome, in Tocantins state; in 2015 the Ministry of Health^2^ reported a change in the pattern of sylvatic YF outbreak dissemination in Brazil in the seasonal period 2014/2015 with 100 confirmed human cases and 51% lethality in the C*errado*, in the midwest states of Goiás and Mato Grosso do Sul, in touristic areas with an intense flow of people in the summer. Fig. 2 illustrates the rapid dislocation of YFV to other Brazilian biomes and recent reintroduction into the Southeast and Atlantic Forest in the ongoing outbreak.

YF epizootics: multiple pathways in viral dislocation

Large epizooties in NHPs have recently been reported in Brazil with 732 YFV laboratory confirmed events in the 2017/2018 monitoring period up to EW 17 (April 28 2018). Epizootics exponentially increased in Brazil in six states (Espírito Santo, Mato Grosso, Minas Gerais, Rio de Janeiro, São Paulo and Tocantins) indicating sustained viral circulation in the pre-seasonal and seasonal periods with São Paulo state accounting for 42% of the total confirmed epizootics.^1^ It is interesting to compare the current outbreak with those in the past. The last severe YFV epidemics in Brazil occurred in 1934-1940, which were comprised of essentially sylvatic cases but also urban transmission with 1,079 confirmed deaths by histopathology.

**Fig. 1:**
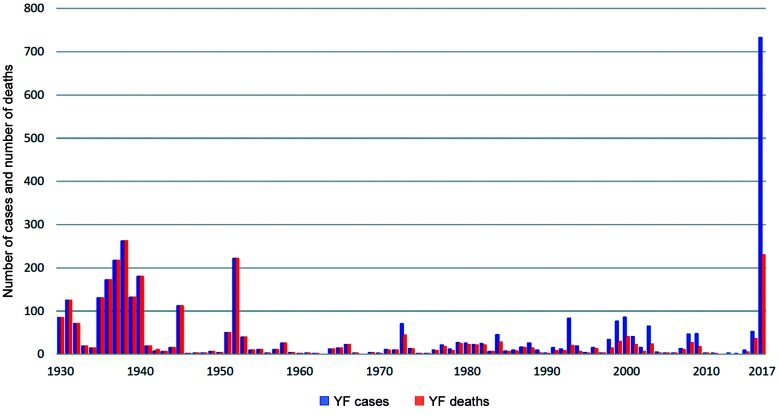
yellow fever: human cases and case fatalities in Brazil 1930-2017. Source: Brazilian Ministry of Health, YF Epidemiological reports 1930-2018.

**Fig. 2:**
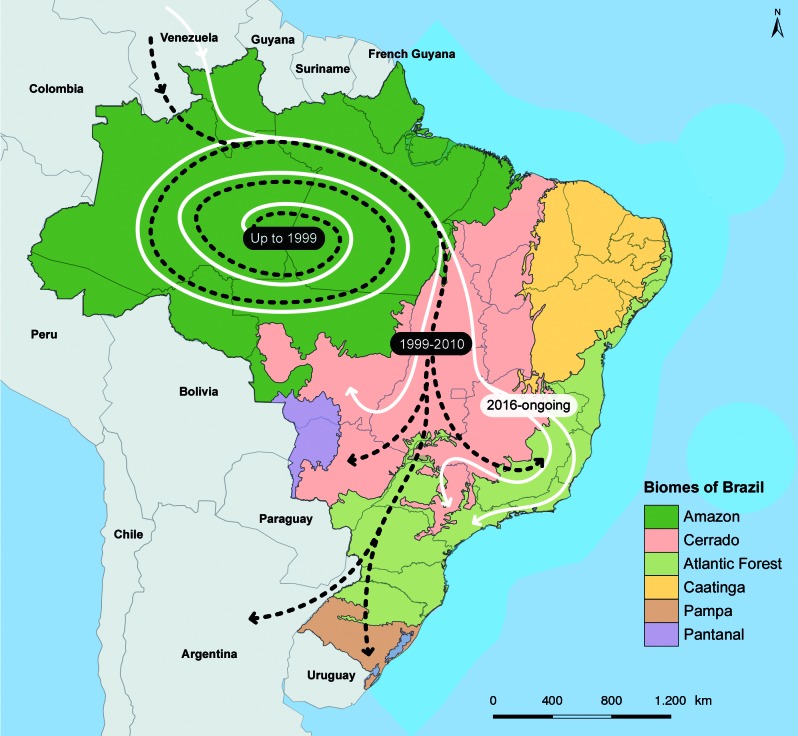
yellow fever virus spread: rapid dislocation into and between Brazilian biomes. Black dashed line: viral spread from late 1980’ until 2010; white line: viral spread from the first half of the 2010 decade onward, including the ongoing outbreak in the Southeast. Sources: IBGE/MMA 2004 for map of Brazilian biomes. Brazilian Ministry of Health//SVS, YF reports from 1999-2018 for epidemiological information on epizootic waves and human cases (http://portalms.saude.gov.br/saude-de-a-z/febre-amarela-sintomas-transmissao-e-prevencao/situacao-epidemiologica-dados).

A comparative analysis contrasting the epidemics of 1934-1940 with that of 2016-2018 allows the identification of important differences in the patterns of YFV spread: (1) the lower human population density in the region in 1934-40 *versus* a much larger population in 2016-2018. The population in the Southeast augmented 368% from 18,345 (19.8 people/km2) to 85,817 thousand inhabitants (92.9/km2) between 1940 to 2015;^3^ ^,^ ^4^(2) still no use of the vaccine for the most part (or totally unused at the beginning) of the epidemic due to unavailability at that time (the first use was in 1937) *versus* availability of a highly effective vaccine produced in Brazil in 2016-2018 which is in contrast to the rapid dissemination of the disease; and (3) underreporting of cases in 1934-1940 since the diagnostic criteria were histopathology the numbers of deaths and cases are the same in the historical tables of Franco^5^ and Benchimol^6^ in 1934-1940 *versus* the use of other diagnostic criteria such as polymerase chain reaction (PCR) in 2016-2018. Fig. 3 shows the route of spatial viral dislocation considering both epizootics and recorded YF human cases during the 1934-1940 in South and Southeast regions^7^ essentially the same touched by the ongoing outbreak. This previous outbreak also followed multiple pathways in epizootic waves originating from the inland *Cerrado* biome (mostly from the states of Goiás and Minas Gerais) and reaching the Atlantic forest biome in the coastal states of São Paulo, Espírito Santo, Rio de Janeiro, similar to that of 2016-2018.^8^ ^-^ ^10^

Although the epizootic waves had propagated in the 1934-1940 outbreak with lower intensity and velocity than in 2016-2018, it is interesting to note the similarities between both outbreaks in spatial dislocation patterns following multiple pathways.

Factors: unusual dislocation and rapidity of YF outbreak

The perplexity with the current epidemiological scenario of the YF outbreak originates from insufficient knowledge on the conditions favoring YFV re-emergence and its very rapid spread from North and Mid-West endemic regions into the coastal Southeastern more populated areas. The dissemination of the YFV as a re-emerging threat in the Americas and the increasing risk of reurbanisation of the disease^11^ ^-^ ^14^ has multifactorial origins and cannot be attributed to just one cause. A modelling study^15^ has examined these multiple factors and the risk of urban YF resurgence in *Ae. aegypti*-infested American cities. Several hypotheses have been considered for the YFV re-emergence in Brazil:

*Viral mutations and evolution* - A recent genome analysis of the circulating YFV in the ongoing outbreak has confirmed that it belongs to the South American genotype I modern lineage and a sublineage, named 1E, which probably independently disseminated from Venezuela into the Brazilian Amazon and then into the inland Centre-West and Southeast (estimated to have taken place around 2005) and South (causing the 2008-2009 outbreak).^16^ The current Brazilian outbreak would be caused by an independent viral dissemination from Northern South America Venezuela into Southeastern Brazil.^10^ ^,^ ^16^ Intriguingly, it was identified that the YFV circulating in the 2016-2018 outbreak has a distinct molecular signature with unique amino acid substitutions mostly located in highly conserved positions in non-structural protein mutations that might affect viral replication impacting the capacity for viral infection in mosquitoes and primate hosts.^8^ ^,^ ^17^ Thus, these viral particularities may have played a role in accelerating the dissemination and severity of the ongoing outbreak.

*Climatic and environmental factors* - Harvard scientists in the 1990’s had already anticipated a dramatic global scenario for the next decades with global warming leading to proliferation and dislocation of mosquito vectors of arboviral diseases, as well as other vectors of infectious diseases, from tropical to temperate zones of the globe.^18^ ^-^ ^20^ More recently, other ecologists of infectious diseases^21^ ^,^ ^22^ have noted that in temperate zones, where the winter temperature is very low for transmission of mosquito-borne infections, warmer summers can trigger the transmission and dislocation of these diseases into new areas, accelerating the parasite development rate and, thereby, making the region more suitable for the transmission of arboviral diseases. Another crucial climatic variable playing a crucial role on mosquito transmitted arbovirus besides temperature is rainfall by supplying suitable larval habitats.^23^ The potential influence of variations in these climate variables in pathogen, vectors and reservoirs in ecological niches together with the possible impact of phenomena such as El Niño, La Niña and ecological disasters are complex and multifactorial, and their potential impact on the environment should be adequately understood. Finally, we emphasize that ecosystems are increasingly being transformed into social ecosystems through human activity. Hence, prevalent theoretical frameworks and eco-social approaches must be reviewed.^24^ ^,^ ^25^ Many factors related to anthropic environmental change, such as the extension of the agricultural frontier resulting from the expansion of agro-industry and other ecological disasters, probably have contributed to this spread. Further research is needed to evaluate the impact of environmental change, which includes new agricultural frontier expansion, on rapid YFV spread in the country. For instance, the so- called “MATOPIBA” territory, which comprises part of the Northeast Brazilian states of Maranhão, Tocantins, Piauí and Bahia, measuring about 400 thousand sq km (equivalent to the territory of Germany), has been the main area of expansion of the agricultural frontier in the last two decades.^26^. MATOPIBA had a huge increase in grain production, mainly soybean, followed by wood devastation and traditionally extensive application of agrochemicals. Possibly, part of the environmental change involving the components of the epidemiological chain of sylvatic YFV may have been affected by such recent agricultural expansion. The potential implications for YF outbreaks of other large environmental disasters in the country, such as the rupture of a mining dam in the city of Mariana, Minas Gerais, in 2015, are still controversial and further research is imperative.

*Mosquitoes: behavior and potential for domestic Aedes transmission* - Climate change, which has most likely led to higher temperatures and rainfall in the recent summer seasons, may have hypothetically favored an increase in mosquito population density in Brazil. Thus, higher mosquito biting rates exponentially increase YFV circulation. The possibility of more efficient YFV vertical transmission in mosquitoes must also be considered. Mondet at al.^27^ have suggested that vertical transmission is one of the key elements in the epidemiology of YFV both in South America and Africa.

**Fig. 3:**
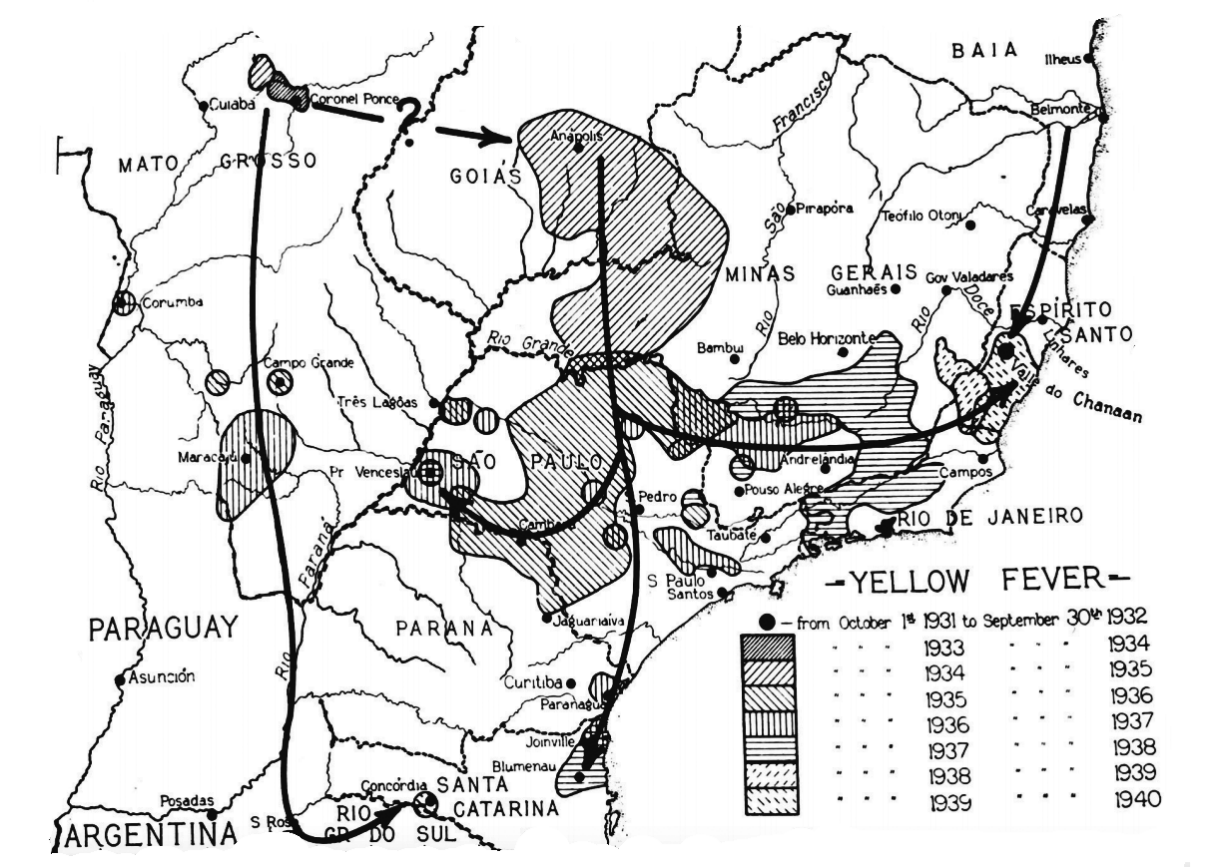
probable route of spread of yellow fever (YF) epizootics of 1934-40 in Brazil as indicated by chronologic appearance of jungle YF. Sources: Benchimol^6^ ^)^ and Strode.^7^

Climatic and environmental conditions also favor mosquito transmission of the virus. Higher average temperatures and rainfall enhance the population density of *Aedini* mosquitoes, such as *Haemagogus* and *Aedes* species, by providing higher availability of larval habitats (mostly tree-holes and bamboo) and accelerating larval development, besides quickening viral replication and dissemination from the mosquito mid-gut to salivary glands. In Southeast Brazil, where the outbreak has spread, the rainy-hot season occurs essentially between November and May, which coincides with the peak of YFV cases and epizooties in 2016-2018.

Concerning the mosquito species vectors of sylvatic YFV in the Southeast, a contrasting preference has been observed according to the vertical distribution in the forest. Two mosquito species have already been confirmed as YFV natural vectors during the ongoing outbreak in Southeast Brazil, *Haemagogus leucocelaenus* and *Hg. janthinomys*.^8^ While *Hg. janthinomys* usually bites in much higher density at the tree canopies, *Hg. leucocelaenus* usually bites more frequently and shelters at the forest ground level.^28^ However, both species may bite both monkeys at the tree canopy and humans at the ground level upon entering or approaching the forest. Although both *Haemagogus* are active all day long, while *Hg. leucocelaenus* has a predominant afternoon biting activity, *Hg. janthinomys* usually reaches a biting peak around midday.^29^ Although primatophilic, *Hg. leucocelaenus* and even *Hg. janthinomys* are eclectic concerning host preference biting other animals such as cattle, birds, dogs, rodents and horses, adapting to the modified environments.^30^

Degradation of natural habitats together with reduction of wild animal population size and consequent lack of blood source for sylvatic mosquito vectors has accelerated recently, increasing human biting and the opportunities for vector sharing between NHPs and human beings. Besides degradation of natural environments, the death of NHPs due to YF may have led mosquitos to seek other blood sources, including humans, in the modified environments, increasing the risk of human infection.

Recently, Evandro Chagas Institute in Brazil has preliminarily reported the finding of YFV natural infection in *Ae. albopictus* captured in 2017 in rural areas near the municipalities of Itueta and Alvarenga in Minas Gerais.^31^ The detection of the YFV genome in a mosquito does not necessarily mean that the insect can play a vector role. Therefore, so far, it is not yet possible to refer to *Ae. albopictus* as a natural vector of YFV. However, *Ae. albopictus* populations from Brazil have proven to be competent to transmit the viruses although with much less efficiency than sylvatic vectors such as *Hg. leucocelaenus* and *Sabethes albiprivus*, simultaneously tested.^32^ Even being apparently poorly competent vectors, domestic and peri-urban Brazilian *Aedes*, such as *Ae. aegypti* and *Ae. albopictus,* may play an important role in YFV transmission given other conditions, such as high vector densities, high human-biting rate, high daily survival rates and low vaccination coverage. Couto-Lima et al.^32^ note that considering all these conditions, it is difficult to understand why YF has not already reestablished an urban cycle in Brazil. Therefore, reducing domestic *Aedes* density in peri-urban and urban areas, with effective control policies^33^ combined with rapid vaccination coverage are essential to avoid YFV transmission in urban centers close to enzootic areas. One recent study^34^ found that *Aedes* are the predominant mosquitoes in nine public parks in the city of São Paulo.

*Environmental policies and human behavior* - Another factor that may help explaining the severity and rapid spread of the ongoing outbreak is the successful environmental and biodiversity protection policies of IBAMA (the Brazilian Agency for Environmental Protection) which has led to a considerable increase in the number of conservation units and legal actions to protect Brazilian fauna with its very strict wildlife preservation measures during the last decades. The total accumulated area held by conservation units in Brazil has been greatly enlarged since the last severe YFV outbreak in the Southeast, consisting of 218,081ha in 1930-1940 to 76,848,771ha by 2009, an increase of 361.5%.^35^ Together, these measures and policies progressively and considerably expanded the areas with environmental and ecological conditions to support mosquito species diversity and abundance as well as the NHP population growth and recolonisation of sites previously reporting extinctions for decades in the Southeast region which has been affected by the YFV since late 2016. Formerly, extensive forests have reduced in size or been altered due to expanding agriculture, livestock and peri-urban growth in the Southeast. But, wood fragments enhanced in size and wild corridors such as gallery forests have gradually recovered during the last decades favoring even more the conditions for proliferation and dispersion of arboreal mosquitoes and NHPs, and consequently arbovirus transmission. Simultaneously and following the example of what has been happening in the world in terms of human way of life and practices, the interest in living in, experiencing and interacting with the natural environment and especially the forest has increased considerably, particularly at the outskirts of the largest southeastern Brazilian cities under influence of the Atlantic forest biome. The vogue of eco-tourism and the rise of some human practices related to the woods such as the so-called back to nature movement, neo-survivalism and sylvotherapy have been reported. Living or having a secondary dwelling in or near the forest became one of the aspirations of contemporary man. These life styles and practices have led people to an increasing approach to the woods and the sylvatic transmission cycles of infectious agents in the Atlantic forest. Therefore, the risk of zoonotic infections has been higher than ever, as illustrated by the malaria outbreaks of simian origin recorded in this biome of Southeastern Brazil in the last years.^36^ Coincidently, YFV and *Plasmodium simium*, the causative agent of these malaria outbreaks, share the same vertebrate hosts, the NHPs, particularly howler monkeys. Yet, these philosophical human approaches to the woods as any other anthropic modification (e.g., agricultural expansion and environmental disasters) result in degradation of original habitats of NHPs and mosquitoes which in turn will be forced to exploit the ecotone between the natural and modified environment and even migrate between patches of woods, favoring viral dispersion. In conclusion, a combination of several factors concerning human behavior and environmental policies have also contributed to recently transform the coastal zone of the Southeast region more receptive and vulnerable to sylvatic YFV transmission and accidental human infection in this cycle.

NHPs: restricted mobility in YFV spread

The susceptibility of NHPs to the YFV is quite variable and seems to depend upon at least two-way interactions between the NHP population and virus strain.^7^ Although susceptible to the YFV infection, African NHPs are resistant to the disease which makes it difficult to isolate the virus from wild-caught animals in the continent where the YFV originated. Indeed, the first isolation of YFV from infected humans could not be achieved in African NHPs until the inoculation of Indian Rhesus macaques.^37^ On the other hand, Neotropical NHP species are in general susceptible to YFV infection and disease. Examining 5,800 Brazilian NHP serum samples for neutralising antibodies, Kumm and Laemmert^38^ reported that immune primates of essentially all genera have been found almost everywhere in the country although antibody prevalence varies not only according to area, but particularly with the NHP genus. A recent study reports the presence of anti-YFV antibodies in howler monkeys (*Alouatta*) captured many months after a YFV outbreak.^39^ Pathogenicity and viraemia (length and viral load) vary according to Neotropical NHP species, which in turn, considerably determines their role as a virus amplifier host in Brazil. Some species may have a prolonged host status for the virus as howler monkeys and marmosets.^40^ ^,^ ^41^ In general, mortality in howler monkeys (genus *Alouatta*) and marmosets (genus *Callithrix*) is high while the capuchin monkeys (*Cebus* and *Sapajus*) seem to have intermediate susceptibility to the disease. Monkey mortality due to YFV in Brazil is considerably concentrated in howler monkeys (*Alouatta*). Their role in the sylvatic cycle is so dominant that their sudden silence and deaths in the woods serves as a signal to local inhabitants and health authorities for the circulation of the virus.^42^ However, marmosets also play an important role. According to Waddell and Taylor,^40^ *Callithrix penicillata* and its hybrids with other marmoset species and even for *Leontopthecus chrysomelas* were very susceptible to the Almada YFV strain in Ihéus, Bahia state. The same authors report that a YFV strain of sylvatic origin was easily maintained by alternate passage through *Callithrix aurita* and *Ae. aegypti* mosquitoes, also achieved with a capuchin monkey species (*Cebus versutus*) though presenting lower mortality.

In the last four decades, the exacerbated degradation of some Brazilian natural environments has accelerated deforestation with the use of fire and big machines, destroying enormous areas of the inland forests. When losing their natural habitats, some NHP species have been forced to relocate and inhabit modified environments and suffer overpopulation of small patches of forest. Altogether, (i) augmentation of conservation areas combined with successful biodiversity protection policies; (ii) high population density of non-immune NHP in the coastal Southeast; and (iii) increased occupation of the modified environment by adapted NHPs such as marmosets and capuchin monkeys in *Haemagogus* infested woods have favored the spread of the sylvatic YFV epizootic waves to this region and consequently, human infection. However, more research is necessary to understand the mechanism by which YFV could travel such vast geographical extensions, so rapidly, as we are witnessing in the ongoing outbreak. It has been suggested that the YFV had an average dispersal of around 3 km per day in the Southeast since early 2017.^8^ ^,^ ^43^ In a previous outbreak in the South, the trajectory of the yellow fever virus circulation was estimated at 600 km in six months.^39^

There is a consensus among experts, in spite of the susceptibility to the YFV infection of some Neotropical genera (e.g., *Alouatta* and *Callithrix*), NHPs are not responsible for such a rapid spread of the YFV. All available knowledge about habits of Southeastern NHP species indicates that their home ranges and daily travelling range in forests are usually restricted, and they normally do not use the ground and deforested areas to migrate from one place to another, except under extenuating circumstances.^44^ Thus, it is highly unlikely that NHPs would carry the infection over long distances. On the other hand, infected mosquitos and humans can disperse the virus over great distances.^12^ ^,^ ^16^ ^,^ ^45^ For instance, sylvatic YFV vectors such as *Haemagogus* mosquitoes may disperse several kms from a releasing point, a contrasting behavior with that of *Sabethes* mosquitoes, secondary sylvatic YFV vectors, where flight is usually restricted to the forest and near surroundings.^45^ ^,^ ^46^ In fact, Causey et al.^45^ demonstrated that females of the mosquito species, nowadays referred to as *Hg. janthinomys/capricornii* and *Hg. leucocelaenus*, could be collected 11.5 km and 5.7 km, respectively, from a releasing forest in Southeast Brazil in few days. During the course of this long displacement, *Haemagogus* mosquitoes may almost continuously traverse large areas of pasture and vast monoculture plantations as well as visit scattered gallery forests and residual forest patches. In other words, these mosquitoes are liable to span long distances beyond the forest where they emerged from their original arboreal larval habitat, a pattern also observed in Central Brazil.^47^ ^,^ ^48^ Of course, this behavior greatly favors continuous viral spread at long distances across these mosquito territories. Female mosquito dispersal is essentially governed by the search for oviposition sites and blood sources, and dispersal advantageously reduces genetic structuring of the population. Several factors may influence *Haemagogus* female mosquitoes from climatic (low rainfall leading to reduction of spatial availability of water-containing tree-holes stimulating dispersal and vice-versa) to environmental and ecological conditions. In the latter two cases, reduction of NHP’s and other wild animal populations’, as a consequence of natural habitat degradation and deaths by YFV, has led to a reduction of blood sources, thus inducing sylvatic mosquito dispersion. In addition to the intrinsic mosquito flight ability, it has been shown that convection currents around twilight aid arboreal mosquitoes to disperse between isolated patches of woods.^7^ ^,^ ^48^ Thus, any factor influencing mosquito dispersal would increase the risk of human biting and infections.

During the ongoing outbreak in the state of Rio de Janeiro two examples of fast and far YFV dispersal can be respectively illustrated by the unexpected sudden human and NHP deaths in the districts of Maricá and Ilha Grande (an island) just after the beginning of the 2017 and 2018 transmission seasons were recorded in the state. The main and large YFV generating foci and dissemination areas in the state has mostly been the denser forests growing on escarpments of the Serra do Mar, a long system of mountain ranges parallel to the Atlantic Ocean coast.^8^ Maricá has considerably small wood fragments poorly connected by scattered remains of gallery forests to the Serra do Mar around 20 km apart, while Ilha Grande is separated from the continent by more than 2 km of sea. It is inconsistent to attribute such efficient dispersions of the YFV to NHPs, whereas the displacement of paucisymptomatic and asymptomatic humans and/or infected mosquitoes may better explain the phenomena. These evidences suggest that NHPs may have a reduced contribution to the rapid spread of the ongoing outbreak.

YF asymptomatic human mobility

Only few YFV infected people become ill and develop symptomatic disease, thus the majority are asymptomatic. Viremic paucisymptomatic or asymptomatic individuals accidentally infected by the bite of sylvatic mosquitoes can travel carrying the YFV and rapidly disseminate the infection to distant receptible areas, i.e., sites infested by competent mosquito vectors. Receptive sites may vary from forest patches, their surroundings, rural areas and microenvironments within urban areas supporting wildlife with propensity to exploit variable environments (or overlap territory with synathropic species) occupied or reachable by *Haemagogus* and/or *Sabethes* mosquitoes as well as peri-urban and urban areas densely infested by *Ae. aegypti* and *Ae. albopictus* populations competent to transmit the YFV*.* The current increasing number of people who move to and from areas of risk in the Atlantic Forest biome has probably favored the rapid spread of the ongoing *Haemagogus* vectored YFV outbreak into urban and peri-urban ecosystems, which has increasingly jeopardised the reemergence of *Aedes* transmission in the peri-urban/urban areas, now a great concern.

Until very recently, YFV was usually acquired by unvaccinated people from the YF free area along the Brazilian coast when visiting the enzootic and epizootic inland *Cerrado* and Amazon biomes for tourism or work. The incidence of such YFV infections had been low, and thus, the risk of reemergence of *Aedes* transmitted YFV in the anthropic environment was reduced before the ongoing outbreak reached the large and highly populated coastal Southeast region. Since the virus and the clinical evolution are identical, there is no distinction between “sylvatic yellow fever” and “urban yellow fever” except for the ecological nature of the transmission, more properly defined by the type of vector mosquito. Currently, even the geographical location of infection acquisition could not suitably define a case as a sylvatic or urban yellow fever event. Indeed, there has been a recent increasing expansion of urbanisation in the Southeast with the continuous construction of urbanised neighborhoods and other types of settlements in areas bordering the enzootic forest attainable by YFV sylvatic mosquito vector flight. Thus, an infection acquired by someone living in a house or visiting a park in an urbanised area near an epizootic patch of wood increasingly deserves a deep epidemiological investigation concerning the origin, since *Haemagogus* mosquitoes may bite far from the wild on even indoors.^47^ In addition, the overlapping territories of confirmed and potential YFV vectors have expanded in Southeast and South Brazil favoring human infection by the bite of sylvatic mosquitoes (*Haemagogus* and *Sabethes*) but also potentialising enzootic spillovers with establishment of YFV either into an intermediate/rural cycle, due to the ecological plasticity of the invader mosquito *Ae. albopictus*, or into a potential urban cycle with limited transmission between the domestic mosquito *Ae. aegypti* and humans (Fig. 4).

In conclusion, although yellow fever viremia is usually short in humans, the possibility of intense mobility of infected but asymptomatic people participating in the virus spread chain is more real than ever, with the increasing overlap between sylvatic and urban ecosystems, in a transition ecological zone (Fig. 4).

The dislocation of asymptomatic YFY viremic people has implications not only for viral spread within Brazil and receptive South American areas but also for global dissemination. The risk of YFV spread in global travels is very high and the reason is that the world has annually higher number of air passengers than ever. According to the UN World Tourism Office, Asia and the Pacific (both infested by *Ae. aegypti*) is expected to witness the strongest growth in tourist arrivals, where the forecast suggests an increase by 331 million over the 2010-30 period to reach 535 million in 2030, with a growth of 4.9% per year.^49^ The recent YF spread via unvaccinated travelers from Angola to China highlights this threat, indicating how individuals can still circumvent the International Health Regulations.^50^ ^,^ ^51^ This scenario highlights the need for permanent international vaccination requirements and molecular surveillance of YFV strains from different regions of the globe.

Response: need to extend vaccine coverage, R & D and production

Historically, increased governmental demands for vaccines have emerged at the peaks of YFV outbreaks or epidemics, which have not been met due to significant vaccine shortages caused by the insufficient federal investments in the main global public manufacturer and exporter of the YF vaccine, Bio-Manguinhos/Fiocruz. This scenario resulted recently in a governmental decision for fractional YF vaccine doses in Brazil, as witnessed in Africa in the last outbreak in Angola, which spread to the Democratic Republic of Congo.^52^ ^,^ ^53^

**Fig. 4:**
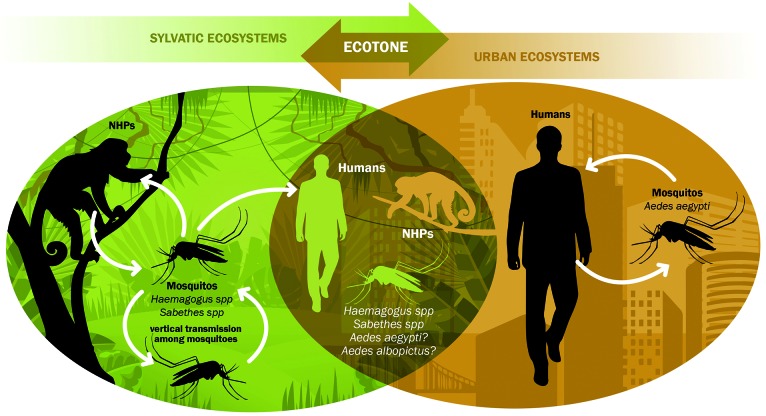
yellow fever virus (YFV) transmission dynamics in Atlantic Forest biome: overlap of ecosystems and ecotone. In the last decades, the progressive combination of several factors has contributed to gradually expand the overlap of ecosystems (sylvatic and anthropic) and the ecotone as well as to imbricate the *Haemagogus-*vectored YFV cycle and the territory of synathropic species, facilitating YFV expansion, human infections and increasingly jeopardised the reemergence of *Aedes* transmission in the urban areas. Among those factors are: (a) the augmentation of areas with environmental and ecological conditions to support mosquito and non-human primate (NHP) diversity and population growth due to the success of recent environmental policies; (b) the loss of some natural habitats forcing NHPs (e.g., marmosets, capuchins) to exploit the ecotone and the modified environments and their overpopulation of remaining forest fragments; (c) adoption of new life styles and practices by the contemporary man leading to an increasing approach to the woods; (d) rapid and intense human displacements; (e) the ecological plasticity of vectors, specially the liability of *Haemagogus* to span long distances beyond the forest limits and *Ae. albopictus* to colonise and disperse from the modified into the sylvatic ecosystem; (f) the crescent infestation rates by *Ae. aegypti* in expanding urban and peri-urban areas near the *Haemagogus*-transmitted YFV cycle.

Availability: YF vaccine shortages and fractional doses

Availability of YF vaccines is a major issue for extending immunisation coverage to the whole Brazilian population. The need to overcome YF vaccine shortages has been historically a concern of Brazilian authorities since the early outbreak in 1988 and later in 2008 when the sudden increase in the demand for YFV vaccines forced Bio-Manguinhos to discontinue exportation to other countries. The Brazilian Ministry of Health enlisted Bio-Manguinhos for a dose-response study with the produced YFV vaccine in order to verify immunogenicity and safety in formulations with lower doses of the 17DD-YFV. The results of this study, conducted by Bio-Manguinhos researchers^54^ indicated that immunogenicity of vaccine formulations with 587 IU or more per dose is similar to the 27,476 IU dose currently applied for short and mid-term protection. There were no differences among groups regarding common adverse events, with exception of pain, found more frequently with the larger dose, reference vaccine. There were no difference among groups regarding proportion or intensity of viremias detected by viral plaque formation or quantitative real-time-PCR (qRT-PCR). Seropositivitity of those who seroconverted was maintained during eight years, with no differences among groups.^55^ A recent study^56^ has advanced evidence base supporting these results.

Considering also the excellent data for vaccine stability observed during the study, it would no longer be justifiable to keep the high dose in current use as a standard in the adult population. Final considerations were adopted, stressing that changes in vaccine formulation would require confirmation of these results in infants, the target population for vaccination in endemic areas. Conclusions of the studies support feasibility of fractional doses as proposed by WHO directives.^57^ Bio-Manguinhos is the only manufacturer using the 17DD substrain in an effort for vaccine sparing. The need to significantly increase Bio-Manguinhos production of YF vaccine to meet this sharp increase in demand for the vaccine from Brazil and other countries is clear. Finally, it is important to highlight that fractional doses of YF vaccine are not yet compliant with International Health Regulations (IHR) and therefore should be used only in outbreak settings until we have more data.

The WHO Global Strategy to Eliminate Yellow fever Epidemics - EYE^58^ supports the need to increase global vaccine coverage and the production of the main manufacturers, such as Bio-Manguinhos. This global strategy has defined goals that should be urgently met by Brazil and other Latin American and Caribbean countries (LAC). One of the key milestones of this strategy establishes that by the end of 2021 all LAC should have completed mass immunisation campaigns. In order to meet this goal, a significant increase in vaccine production and availability must be provided by Brazil and other countries in the LAC region. The global supply of YF vaccines is expected to increase to between 116 and 159 million doses in 2021, and to between 162 and 183 million doses in 2026. The increased capacity expected during 2017-2020 should be achieved by prioritising and optimising YF vaccine production and contracting manufacturer operations for filling and freeze-drying. New production capacity is mainly expected after 2021, when new facilities in two manufacturers will start production.

Coverage issues: adverse effects and vaccine refusal

A recent study^59^ has provided an overview of global YF vaccination coverage from 1970 to 2016, based on adjusted retrospective analysis, indicating the urgent need to increase global coverage of the YF vaccine. It was estimated that between 303.7 million and 472.9 million people leaving in areas at risk of YFV transmission still require vaccination to achieve the 80% population coverage threshold recommended by WHO. This represents 43-52% of populations in YF zones, compared with the population who would have required vaccination in 1970. Nevertheless, it is important to note that besides economic and policy constraints, there are behavioral obstacles to meet this goal. One of the main reasons is that individuals feel safe when they are in urban and peri-urban areas or even in rural zones when they do not enter the forest. Even if they work or recreate on the open fields attend clubs, resorts and parks, in or outside the forest ecotone, their perception of risk of acquire the YFV is very low, because they are usually not convinced that they are potentially exposed to YFV infected mosquito bites (Fig. 4). This perception of low risk combined with their exacerbated perception of adverse effects of YF vaccine consists of an important hindrance for the achievement of the optimal vaccination coverage.

On 25 January 2018, Brazilian health authorities began a vaccination campaign (fractional and standard doses) targeting 69 municipalities in Rio de Janeiro (10 million persons in 15 municipalities) and São Paulo (10.3 million in 54 municipalities) states. As of 16 February 2018, preliminary results of the mass YF vaccination campaign indicated that 4.3 million persons had been vaccinated for YF (3.9 million persons with fractional doses and 379,987 persons with standard doses).^60^ This represents 21% of the planned coverage of over 20 million people targeted for vaccination in the two mentioned states. Due to the low vaccination rate achieved during the campaign in Rio de Janeiro state (12.1% of the targeted population), state health authorities extended the campaign. Similarly, in São Paulo state, only 29.6% of the targeted population had been vaccinated during the campaign and state health authorities are assessing whether or not to extend the campaign.^61^ ^,^ ^62^ To widen this very low vaccine coverage in the campaign, the states of Rio de Janeiro and São Paulo decided to continue the population vaccination strategy with support of the Ministry of Health. Preliminary YF immunisation data sent to the Brazilian Ministry of Health by the Health Secretariats of the states of Bahia (55% of target population), Rio de Janeiro (71% of target population) and São Paulo (90% of target population) indicate that this coverage has significantly improved since the beginning of the campaign with 17,300,000 people vaccinated. But the coverage is still low and additional efforts and more proactive initiatives should be taken with support of the educational system and more effective media communication strategies to extend YF vaccine coverage to the whole Brazilian population. It is important to note that one of the main issues for this low vaccination rate are not only related to the availability of YF vaccines but to vaccine refusal, particularly in more affluent groups, related to exacerbated perception of the vaccine adverse effects from erroneous information disseminated through social media and networks in the internet as well as misunderstanding of the effectiveness of fractional doses.

Immunisation of NHPs: monitored areas

The immunisation of NHPs should also be a priority for animals confined in parks and forest reserves contiguous to urban areas as well as animals maintained in captivity in research centers and zoos where the monkey population is monitored and thus immunisation feasible. Immunogenicity studies with the 17D vaccine have only been conducted in the Indian Rhesus macaque.^63^ Antibody response in monkeys inoculated with graded doses of the 17D vaccine was subject to pioneer studies in the 1970’s.^63^ ^,^ ^64^ No similar studies on howler monkeys or any other very susceptible and efficient amplifier hosts have ever been carried out. Although it is impossible at this stage to recommend 17DD / 17D vaccination of howler monkeys and other NHPs, there is an urgent need to accelerate safety and immunogenicity studies with the 17D vaccine in such Neotropical hosts, especially howler monkeys, which should be expedited in regulatory procedures due to the YF emergency. Although the experimental immunisation of monkeys would greatly diminish the sentinel role NHPs play in such selected confined areas, it would significantly reduce viral circulation in risk areas besides preserving biodiversity and protecting endangered species in conservation areas. In fact, local NHP populations may be extinct or greatly reduced as a consequence of devastating YFV epizooties.^7^ ^,^ ^65^ ^,^ ^66^ Although controversies among primatologists on the feasibility of NHP immunisation have come to light, considering potential difficulties in capture and/or vaccine injection in free-living animals in the open fields due to their high mobility, novel technological strategies for immunisation should be conceived and evaluated in Brazil. Many potential technological approaches could be adopted for such immunisation such as food containing recombinant vaccine, transgenic fruit and other technological alternatives. Of course, these possibilities must be very well discussed with people involved in the preservation of NHPs, environmental experts, scientists and governmental bodies for an adequate financial support. A pilot study with existing 17DD YF vaccine in Golden-headed lion tamarins - GHLT (*Leontopithecus chrysomelas*) and howler monkeys (*Alouatta sp*) is currently in progress at Bio-Manguinhos/Fiocruz in collaboration with the Rio de Janeiro Primatology Center. Although YF has not sufficiently been studied in Neotropical NHPs and there is no current proven vaccination for NHPs, there are operational difficulties to apply traditional needle immunisation strategies in this population due to high monkey mobility. It is therefore necessary to advance and develop the above mentioned novel monkey immunisation delivery strategies. This type of initiative must be supported by awareness of the beneficial role of NHPs as a sentinel of YFV circulation and not as direct disseminators of the YF etiological agent harmful to the human population. During the 2008-2009 YFV outbreak in Rio Grande do Sul, South region, misinformation resulted in aggression towards monkeys and even extermination^67^ leading to the local red howler to be once again listed as an endangered species in Brazil. A similar situation has occurred in the Southeast region where some of the most endangered NHP species living on the continent have already been greatly reduced in the Atlantic Forest. In fact, misinformation has created a negative image of the NHP in public opinion.

Final considerations

For decades, Brazilian scientists have been warning the public health authorities that once the virus started to circulate in the Atlantic Forest biome close to large metropolitan areas, the risk of reurbanisation of a disease, so far limited to the sylvatic cycle in the endemic/enzootic Amazon Forest and the inland *Cerrado* biomes, would become more real than ever. The recent territorial dislocation of the YFV has not been linear. As in previous large sylvatic outbreaks it has taken place in complex and multiple pathways, in epizootic waves combined with the unprecedented intense mobility of asymptomatic infected humans involved in rural work, eco-tourism, extrativism and animal traffic. A second reason for this concern is the possibility of a catastrophic event affecting a huge number of non-vaccinated people as confirmed by the current outbreak. The complex epidemiological patterns of the disease dissemination have detrimentally combined with the very low vaccination coverage of the population in the main metropolitan areas, which persists even after the ongoing YF outbreak, together with the inefficient *Ae. aegypti* control measures in peri-urban and urban areas. Moreover, despite recent advances in understanding the eco-social determinants involved in the rapid spread of the ongoing outbreak, several knowledge gaps still persevere. The hypothesis of the possible role of asymptomatic infected humans in YF outbreaks in Brazil requires further research. As well, the mechanisms underlying the rapid spread of epizootics in the country must be understood. Epidemiological parameters to provide support to these hypotheses will be necessary. Many issues related to molecular epidemiology and transmission cycles of YFV in the diverse Brazilian biomes remain poorly understood. Support for research and development activities in this field is urgently needed, promoting innovative governance strategies with targeted investments aiming to improve surveillance and fast response to the epidemics. Immunisation coverage of the Brazilian population living in the affected areas should urgently target the entire population in order to minimise the impact of the ongoing outbreak.^68^ Novel monkey immunisation strategies are also crucial in parks, preservation areas and zoos, avoiding extinction of species at risk and preserving biodiversity. Finally, it is imperative to continuously monitor YFV receptivity and vulnerability conditions, strengthening the National Laboratory Network for YF diagnosis, in order to improve the quality of response and prevent future outbreaks possibly leading to catastrophic reurbanisation of the infection in Brazil and other Latin American and Caribbean countries.
